# Obituary: Alan Raybould

**DOI:** 10.3389/fbioe.2022.1124965

**Published:** 2023-01-12

**Authors:** Lawrence Dritsas, Joyce Tait, Geoff Simm, Geoffrey Banda, Mike Bowes, Jim M. Dunwell, Karen Holt, Theo Papaioannou

**Affiliations:** ^1^ Science Technology and Innovation Studies, Global Academy of Agriculture and Food Systems, University of Edinburgh, Edinburgh, United Kingdom; ^2^ UK Centre for Ecology and Hydrology (UKCEH), Wallingford, Oxfordshire, United Kingdom; ^3^ School of Agriculture, Policy and Development, Department of Crop Science, University of Reading, Reading, United Kingdom; ^4^ Syngenta, Cambridgeshire, United Kingdom; ^5^ Innogen Institute, Department of Development Policy and Practice, Open University, Milton Keynes, United Kingdom

**Keywords:** Raybould, biosafety and biosecurity, Syngenta, CEH, Edinburgh

Professor Alan Raybould was born and raised in Wolverhampton, in the West Midlands, United Kingdom. He attained a First Class Degree in Botany from the University of Manchester, followed by his PhD in Population Genetics at the University of Birmingham in 1989, researching population genetics of *Spartina anglica.* Alan began his scientific career at the Institute of Terrestrial Ecology at Furzebrook, Dorset, which later became part of the United Kingdom Centre for Ecology and Hydrology. During this period (1990–2001), he progressed from a post-doctoral research position to becoming the lead scientist in molecular ecology, studying gene-flows from genetically-modified crops to related wild plant populations. He joined Syngenta in November 2001, and worked in the Product Safety department, leading the preparation of environmental risk assessments as part of Syngenta’s global regulatory submission for transgenic crops.

At Syngenta, Alan was proactive in developing new paradigms for risk assessments and proposing new regulatory frameworks in which products could be effectively assessed. His work in this area was recognised internationally and had a profound effect on the way environmental risk assessment of genetically modified crops are conducted. He was an active contributor to the Society for Biosafety Research (ISBR), where he helped in the programme committee and editorial board. Alan also actively participated and contributed to the organisation of many international scientific conferences and courses. He became well-known for his clear thinking and ability to explain complex concepts in a practical and understandable way. Alan was a prolific writer and author on a large number of publications. He also took on roles for scientific journals such as that of Specialty Chief Editor for *Frontiers in Bioengineering and Biotechnology*. He was also a member of the Editorial Board of *GM Crops and Food*. To reflect his international influence, Alan was appointed to a Syngenta Fellow position in Syngenta and continued to be a regular invited publisher and speaker on the environmental risk assessment of transgenic crops. It was in this advocacy role that Alan first worked closely with colleagues at the Innogen Institute, a collaboration between the University of Edinburgh and the Open University, because he was the main Syngenta contact and partner in a joint ESRC/Syngenta-funded knowledge exchange project “Engaging with Uncertainty and Risk in Agricultural Biotechnology Regulation”. He continued to stay in close contact Edinburgh colleagues after the project was completed.

In 2019, Alan was appointed to the Chair of Innovation in the Life Sciences at the University of Edinburgh. This important professorship was jointly held between Edinburgh’s Science Technology and Innovation Studies subject group (STIS) and Global Academy for Agriculture and Food Systems (GAAFS). At Edinburgh, Alan embraced his new role as an educator. He was a co-organiser of the undergraduate course Innovation in Sustainable Agri-Food Systems, co-organiser on the postgraduate course Innovation in Sustainable Food Systems and had designed his own postgraduate course Making Science Relevant to Policy and Decision-making, which after just 1 year of delivery had gained in popularity amongst students. He was delighted to supervise PhD and Masters students across STIS and GAAFS. In research, Alan made important contributions in consolidating and extending Innogen’s work into the regulatory and commercial challenges of novel agricultural technologies, whilst building important and enduring links with GAAFS. He brought a rich blend of industry, policy, and science experience to all his work. It is not surprising that in a short time with colleagues in the Innogen Institute and GAAFS in Edinburgh University, they had managed to get a number of industry-academia collaborations started.

In early 2020, Alan successfully applied to join the Advisory Committee on Releases to the Environment (ACRE), an advisory public body sponsored by the UK’s Department for Environment, Food and Rural Affairs. In this role, Alan was one of a small group of experts who provide statutory advice to government ministers on the risks to human health and the environment from the release of genetically modified organisms (GMOs). Before falling ill, he was set to become vice-chair of the committee.

Alan was a gentle, fun and approachable colleague who brought a rich blend of industry, policy, and science experience to all his work. He was a much loved and respected member of the academic community who will be sorely missed by his colleagues and students.

